# Supplementary material for: Representation of professions in entertainment media: Insights into frequency and sentiment trends through computational text analysis
Source: PLoS One. 2022 May 18;17(5):e0267812. doi: 10.1371/journal.pone.0267812 (PMC9116627; doi:10.1371/journal.pone.0267812)
Supplement: Attachment — Submitted filename: PLOS Response to Reviewers.pdf [file pone.0267812.s002.pdf]

Dear Editor and Reviewers,

Thank you for your thoughtful feedback on our manuscript titled "Representation of professions in entertainment media: Insights into frequency and sentiment trends through computational text analysis". They have helped us to improve the paper significantly. Please find our response to your comments below.

The editor noted that grant information in the ‘Funding Information’ and ‘Financial Disclosures’ sections do not match. This is because our work was supported by a gift grant which did not have a grant number. We have added the U.S. Chamber of Commerce Foundation as a funding source without specifying a grant number in the revision. Our financial disclosure statement is: "The study was done at Signal Analysis and Interpretation Laboratory, University of Southern California, which is supported by a research award from the U.S. Chamber of Commerce Foundation (https://www.uschamberfoundation.org/). The funders had no role in study design, data collection and analysis, decision to publish, or preparation of work included in this submission." As per the editor’s suggestion, we have included the updated financial disclosure statement in the cover letter as well.

We have also included a section below called “Editor(Email)” below to respond to the paraphrasing suggestions raised by the editor in the email sent on Mar 7th with the subject: PLOS ONE Decision: Revision required [PONE-D-21-27904R1] - [EMID:931db3d1d60343a8].

Sabyasachee Baruah
Krishna Somandepalli
Shrikanth Narayanan

Editor (Email)

Comment

In response to me and Reviewer 1, you have included some suggestions as to factors that might be responsible for the changes that have been detected in the corpus. Clearly these are speculative, as we suggested they might be, but I think some further text is necessary to draw attention to the speculative nature of the proposed connections, and the need for further work to establish whether or not they actually hold.
My suggestion would be to insert some text at the start of the Discussion section, noting that you will tentatively suggest some possible causes of the changes. This might allow you to deviate a little from the "X might be because of Y" template you followed throughout the discussion, which I found a little repetitive. I think it would be more satisfactory to employ forms of language like "There is some evidence of Y [reference], which could lead to [X happening]" and variants that remove repetition. Where possible it could be useful to add comments as to

the mechanism by which it might filter through to your dataset and/or what you might need to do in practice to demonstrate the connection.

Response

We changed the introductory passage in the discussion section to emphasize that we selected a few interesting trends to discuss and our hypotheses to explain these trends are purely speculative. We also state that verifying our hypotheses would require further research and controlled experiments. As of now we are still unsure what kind of future experiments we would need because it is difficult to design studies that can establish any form of relationship between media narratives and real-world societal factors. Nevertheless, we suggest some ideas in our discussion.

We have also created a visualization tool that allows users to explore the trends in our subtitle corpus. We cite it in the discussion section. Also, as per the editor's paraphrasing-related requests, we have varied the language to deviate from the "X might be because of Y" template.

Revision

Lines 704-715

The frequency and sentiment expressed towards professionals vary in media content depending on the character archetype, the story being told, year of production, and genre, among other variables. We developed a visualization tool [75] to examine the frequency, sentiment, media attribute, and employment trends in our subtitle corpus. While examining the wealth of results using this tool, a few general findings and specific trends stood out to us. In this section, we highlight these trends, discuss some reasons that may be suggestive for explaining the observed results, and try to reconcile these findings with those in relevant studies, both from the real world and the media domain. We underscore that our interpretation is purely speculative and requires careful, controlled experiments and surveys to further validate the claims. Also, this discussion is by no means an effort to explain all our findings, and we hope future research in this space may further explain some of the results we found in this large-scale analysis.

Lines 718-720

We suggest that this trend is due to the increased awareness stemming from conversations about gender-neutral terms among the youth, parents, and the LGBTQ+ community [76, 77]

Lines 734-736

These advances and interests potentially explain the increase in the frequency of STEM-related and sports portrayals on the big screen.

Lines 741-751

We posit that as new and specialized professions become popular career choices and essential in catering to the needs of the continuously evolving society, they also attract the interest of media creators. Technology improvements in computerized financial transactions (barcode scanning, automatic teller machines, and mobile banking) and customers' preference for self-service checkouts probably contributed to the decrease in employment of bankers and cashiers [84, 85], or at least their explicit mentions in media narratives.

Lines 773-781
We have made several hypotheses to explain the observed frequency and sentiment trends and require controlled experiments to verify these claims. It is challenging to design such studies because we are trying to relate media narratives to real-world events – two very different domains. As of now, we are unsure regarding what types of experiments are required, but we suggest some ideas. We can survey scriptwriters and movie directors to find what societal events inspired their stories and creative decisions. We can also try to map change points in media trends to real-world events to explain their possible cascading effects on media narratives and genres. We leave this task for future research.

Editor

Comment 1

Both reviewers agree that this is a worthwhile and carefully conducted study, something that I also sensed when reading the manuscript. All of us appreciate the clear writing style.

Response

Thank you.

Comment 2

Reviewer 1 raises some "high-level" issues, mostly around the interpretation of your findings. They are particularly concerned about possible causal relationships between the correlates that you identify in your work, and I agree that this needs some finessing in a revision. As Reviewer 1 states, if one wishes to offer a particular causal interpretation, evidence needs to be provided to support this; otherwise, one should instead emphasize the nature of the correlations and at most tentatively suggest causal hypotheses that could be further explored in future work.

Response

We agree with the editor and reviewer 1, and acknowledge that we should refrain from causal assertions in our correlational study. We replace all potential ambiguous/ misleading statements like "media is a mirror to society" with "media and societal trends correlate with each other". While establishing causality would have been a much stronger result, showing that with our present experimental setting is difficult without accounting for additional demographic, political, technological, and cultural factors along with establishing the ecological validity of such findings given the constraints of the confounding variables. Our effort is a step towards this direction, and future work should include more controlled studies. We highlight this in our updated conclusion and discussion sections.

Revision

Abstract

Professions that employ more people ~~have~~ showed increased media frequency, ~~supporting our hypothesis that media acts as a mirror to society.~~

Lines 686-688

In this section, we ~~test the hypothesis that media stories reflect real-world events~~ study the correlation between media frequency and real-world events, namely employment figures.

Lines 697-698

Therefore, the trend of media frequency ~~mirrors~~ correlates with the employment trend for most SOC groups.

Lines 798-802

We observed a significant positive correlation between media frequency and employment trends of most SOC groups. Professions that typically employ more people were also more frequently mentioned in media content. We note that these correlation findings do not imply causality; a question that requires further systematic study.

Lines 831-832

Importantly, future work should also consider investigating causal relations, beyond correlations, between media representations and employment trends.

Comment 3

At the same time, Reviewer 1 notes that the manuscript presents a body of facts, but does not offer possible explanations for them - so clearly some speculation is to be appreciated, as long as it is presented as such.

Response

We have reworked the discussion section to suggest possible reasons behind the observed media frequency and sentiment trends. We could not always find proper references to support our hypotheses, and sometimes even found published studies contradicting our media trends. We have included the relevant published work and have tried our best to explain the media trends.

Revision

Lines 717-720

We observed that gender-neutral terms like massage therapists and flight-attendants are becoming more frequent than their gendered counterparts. We suggest that this trend is due to the increased awareness stemming from conversations about gender-neutral terms among the youth, parents, and the LGBTQ+ community [76, 77].

Lines 720-727

Analyzing explicit gendered profession terms, we observed that the frequency of some female job titles such as waitresses, congresswomen, and policewomen has either increased or remained steady relative to the corresponding male job titles (i.e., waiters, congressmen, and policemen). Equal opportunity legislation, women's rights movements [78], and constitutional amendments for women's suffrage [79] have improved the representation of women in police and government. This may have driven a similar trend of increasing female representation in media content

Lines 730-736

The frequency of STEM, sports, arts, and design occupations has increased, whereas the frequency of construction, farming, and manual labor jobs has decreased. Improvements in cinematography and visual effects technology, space travel, the emergence of the world wide web, and sports documentaries have contributed to the increased fascination of science and sports in media [80, 81]. These advances and interests potentially explain the increase in the frequency of STEM-related and sports portrayals on the big screen.

We have similarly revised the entire discussion section and offered possible hypotheses to explain the observed media trends.

Reviewer 1

Comment 1

Abstract: "Professions that employ more people have increased media frequency, supporting our hypothesis that media acts as a mirror to society." -- Let E be the levels of employment in various professions. Let F be the frequency of various words for professions. Let S be the sentiment of various words applied to professions. The sentence quoted here suggests that variation in E causes variation in F and S: that is, E is the real thing, whereas F and S are merely reflections (mirrors) of E. The paper makes it clear that E is correlated with F and S, but the paper does not demonstrate how the causal connection goes. There are (at least) three possibilities: (1) Variation in E causes variations in F and S. (2) Variations in F and S cause variations in E. (3) Variation in some other thing, X, causes variation in E, F, and S. All three of these causal connections seem possible to me, and it is not at all clear which is the actual connection. The authors need to either (A) be clear that they have only demonstrated correlation and not causation or (B) provide solid evidence that there is indeed a specific causal relation connecting E, F, and S. If the authors choose (A) (causation unknown), then they should remove all expressions, such as "acts as a mirror", that imply a specific causal relation. My guess is option (3): changes in technology (X) drive changes in the level of employment in various professions (E), they drive changes in sentiment towards various professions (S), and they drive changes in the frequency of various words for professions. But I have no rigorous evidence for this hypothesis.

Lines 676-677: "In this section, we test the hypothesis that media stories reflect real-world events."
-- The word "reflect" here is somewhat metaphorical. There is an implication that real-world events cause media stories. But perhaps media stories cause real-world events. Or perhaps both are caused by some third factor. All that the authors can say with certainty is that there is correlation. I would very much welcome some explicit discussion of causality in this paper, but I don't think it's right to smuggle causality into the paper using terms such as "reflect". If the authors are unable to firmly state that there is a certain causal connection, they could still offer some tentative hypotheses about causation.

Lines 726-720: "Lastly, we observed that media frequency correlates with the employment trend of most SOC groups. Professions that employed more people were also more frequently mentioned in media content. This supports our hypothesis that media mirrors society and plays a role in our professional choices."
-- Again, the word "mirror" suggests that society is the cause and media is the effect. This paragraph starts with "correlates" (quite rightly) and ends with "mirrors" (somewhat unscientific). I encourage some discussion of causality, but it should be explicit and straightforward, acknowledging the difficulty of causal analysis.

Response

We agree that correlation between E and F (or S) does not imply causation. It was not our intention to suggest so either. Therefore, we have rephrased all statements relating media trends and employment to explicitly contain the word “correlation”, and removed misleading statements like “media is a mirror to society”.

Revision

Same as the revision to the Editor's second comment (see above).

Comment 2

Lines 8-10: "Cultivation theory suggests that prolonged exposure to the content we see on TV shapes our outlook and makes us believe that to be our reality [1]."
-- There should be some discussion of alternatives to Cultivation theory. For example: The Fruits of Cultivation Analysis: A Reexamination of Some Effects of Television Watching (pp. 287-302), Michael Hughes.
-- https://www.jstor.org/stable/2748103
-- Wikipedia says that there are two alternatives to Cultivation theory that are more widely used: "In a 2004 study, surveying almost 2,000 articles published in the top three mass communication journals since 1956, Jennings Bryant and Dorina Miron found that cultivation theory was the third most frequently utilized cultural theory."
-- https://en.wikipedia.org/wiki/Cultivation_theory

Response

We have provided some discussion on alternatives to Cultivation theory. This debate surrounding the relationship between television viewing and human behavior and beliefs suggests that we require better designed and more demographically inclusive surveys. We have added the suggested citations for completeness.

Revision

Lines 8 - 15

Cultivation theory suggests that prolonged exposure to the content we see on TV shapes our outlook and makes us believe that to be our reality [1]. Several social studies have explored the application of cultivation theory and confirmed its validity [2–4]. However, there are social scientists that have also questioned the validity of the cultivation theory because it ignores socioeconomic factors [5], living conditions [6], and differences in the portrayal of violence on television [7]. Still, this discussion about television and the cultivation of beliefs inspires us to examine their relationship

Comment 3

Lines 553-557: "The frequency trend of the SOC group does not reflect the frequency trend of all its professions. For example, Figs 8 e) and f) show increasing trends for many sales-related professions, but the overall frequency of the Sales Occupations SOC group decreased. A large proportion of Sales Occupations mentions are comprised of bankers and cashiers, whose frequencies have decreased."
-- Do the authors have an explanation for this? The paper should not be merely a collection of facts. Perhaps it is difficult to prove an explanation is correct, however it would be useful to suggest some hypotheses.

Lines 569-570: "Therefore, the number of positive sentiment mentions is greater than the number of negative sentiment mentions for all the SOC groups."
-- Again, some hypothetical explanations would be of interest to the reader.

Lines 589-598: "Fig 12 shows the Spearman's rank correlation coefficient [...] also one of the most positive sentiment trends."
-- Some hypothetical explanations would be of interest to the reader.

Response

Similar to the response to the Editor's third comment, we have included possible hypotheses with relevant citations to explain the observed frequency and sentiment media trends. The analysis section contains the facts and the discussion section provides possible reasons for these facts.

Revision

Same as the revision to the Editor's third comment.

We summarize our edits for the low level comments raised by reviewer 1 here:

1. We replace "like" with "such as" at the suggested places.
2. We replace "if" with "whether" at the suggested places.
3. We replace URLs with proper citations for the profession corpus, sentiment dataset, and sentiment algorithm.
4. We replace "males" and "females" with "men" and "women" respectively.
5. We expand the abbreviated forms of CRF and TAC KBP.
6. We describe the LCF BERT model in simpler terms.
   Lines 386-391

The LCF model defines a value called semantic relative distance (SRD) for each word in the sentence. SRD is the absolute difference between the word position and the target (professional mention) position in the sentence. The model masks or weighs down the output features of words whose SRD is larger than some threshold, that is, the model reduces the effect of words that are farther away from the profession word in determining the sentiment.

Reviewer 2

Comment 1

Although the data and methodology are clearly presented, I would have appreciated the authors pointing out more explicitly their limitations….Also, it would be relevant to underline the main limitations of the study and to provide recommendations for future research in computational social sciences especially in other national and linguistic contexts.

Response

We acknowledge that the earlier version of the manuscript lacked a discussion of the limitations of our work. While ours is the first large-scale computational analysis on the representation of professions in media subtitles, we can further improve our methods through more sophisticated modeling. Moreover, our domain choice limits the information we can glean about the media portrayal of professions. We have described these limitations and more at the end of the discussion section.

Revision

Lines 803-820

There are several limitations of the present work. First, subtitles provide only a limited view of the complete movie or TV show. Features, such as the character's behavior on screen, their interactions with other people, and their character arc can only be studied by carefully viewing the entire movie or TV episode. Therefore, our representation measures of frequency and sentiment of job titles in subtitles do not capture all aspects of their media portrayal. Second, our frequency and sentiment analyses do not control for the frequency or sentiment expressed toward these professions in everyday language. For example, the frequency of policewomen might have been less than the frequency of policemen in media subtitles because that is how their usage evolved in literature. A possible solution is to use the corresponding Google ngram frequencies [23] of the professional words as covariates in our analysis. Third, our study has primarily focused on the representation of professions in the US and the UK (see the distribution of production country in section OpenSubtitles). One needs to adapt our proposed taxonomy and models for different cultures and languages. Our profession taxonomy

does not contain all job titles in other countries. Depending on the application of this taxonomy, one may have to add professions specific to a local region for a comprehensive study.

Comment 2

The discussion section need substantive rework. While it summarizes well the main results of the article, it fails to engage in a reflective discussion with the findings of other studies. The discussion part would benefit from citing more social sciences references supporting the idea that "media mirrors society and plays a role in our professional choices".

Response

We have reworked the discussion section extensively and provided possible reasons behind the observed media frequency and sentiment trends of professions.

Revision

Please see the revision to the Editor's third comment.

References

We have not removed any previous references from our manuscript. We have cited the following new references:

5. Hughes M. The Fruits of Cultivation Analysis: A Reexamination of Some Effects of Television Watching. The Public Opinion Quarterly. 1980;44(3):287–302.

6. Chandler D. Cultivation Theory; 1995. http://visual-memory.co.uk/daniel//Documents/short/cultiv.html.

7. Newcomb H. Assessing the Violence Profile Studies of Gerbner and Gross: A Humanistic Critique and Suggestion. Communication Research. 1978;5(3):264–282. doi:10.1177/009365027800500303.

26. Baruah S, Somandepalli K, Narayanan S. GitHub repository: Representation of professions in entertainment media; 2022. Available from: https://github.com/sabyasachee/mica-profession.

44. Princeton University. WordNet lexicographer file names and numbers; 2010.

https://wordnet.princeton.edu/documentation/lexnames5wn.

75. Chaput M. GitHub repository: Whoosh; 2018. Available from: https://github.com/mchaput/whoosh.

78. Berger M. A guide to how gender-neutral language is developing around the world; 2019. https://www.washingtonpost.com/world/2019/12/15/guide-how-gender-neutral-language-is-developing-around-world/.

79. Williams D. The Rise of Gender Neutrality and its Impact on Language; 2018. https://www.translatemedia.com/us/blog-usa/rise-gender-neutrality-impact-language/

80. Nicholson P. The second wave: A reader in feminist theory. Psychology Press; 1997.

81. Brown JK. The Nineteenth Amendment and women's equality. The Yale Law Journal. 1993;102(8):2175–2204.

82. Geraghty L. American science fiction film and television. Berg; 2009.

83. Jenns N. Why Suddenly Real-Life Sports Documentaries Trend in Film Industry?; 2021. https://www.tetongravity.com/story/news/why-suddenly-real-life-sports-documentaries-trend-in-film-industry.

84. Hauer KE, Durning SJ, Kernan WN, Fagan MJ, Mintz M, O'Sullivan PS, et al. Factors Associated With Medical Students' Career Choices Regarding Internal Medicine. JAMA. 2008;300(10):1154–1164. doi:10.1001/jama.300.10.1154.

85. Dalen JE, Ryan KJ, Alpert JS. Where have the generalists gone? They became specialists, then subspecialists. The American journal of medicine. 2017;130(7):766–768.

86. Reimink T. Are Bank Tellers and Retail Cashiers Experiencing Similar Transformations?; 2017. https://www.linkedin.com/pulse/bank-tellers-retail-cashiers-experiencing-similar-timothy-reimink/.

87. Wong JC. End of the checkout line: the looming crisis for American cashiers; 2017. https://www.theguardian.com/technology/2017/aug/16/Retail-industry-cashier-jobs-technology-unemployment.

88. Flanagan TJ, Vaughn MS. Public opinion about police abuse of force. Police violence: Understanding and controlling police abuse of force. 1996;30(5):397–408.

89. Funk C, Hefferon M, Kennedy B, Johnson C. Trust and mistrust in Americans'

views of scientific experts. Pew Research Center. 2019;2:1–96.

90. Doherty C, Kiley J, Daniller A, Jones B, Hartig H, Dunn A, et al.. Majority of public favors giving civilians the power to sue police officers for misconduct; 2020.

91. Kennedy BR, Mathis CC, Woods AK. African Americans and their distrust of the health care system: healthcare for diverse populations. Journal of cultural diversity. 2007;14(2).

92. Ramakrishna A, Martinez VR, Malandrakis N, Singla K, Narayanan S. Linguistic Analysis of differences in portrayal of movie characters. In: Proceedings of the 55th Annual Meeting of the Association for Computational Linguistics (Volume 1: Long Papers). Vancouver, Canada: Association for Computational Linguistics; 2017. p. 1669–1678. Available from: https://aclanthology.org/P17-1153.
